# Symptoms of posttraumatic stress partially mediate the relationship between gender-based violence and alcohol misuse among South African women

**DOI:** 10.1186/s13011-023-00549-8

**Published:** 2023-06-22

**Authors:** Kim A. Nguyen, Bronwyn Myers, Naeemah Abrahams, Rachel Jewkes, Shibe Mhlongo, Soraya Seedat, Carl Lombard, Claudia Garcia-Moreno, Esnat Chirwa, Andre P. Kengne, Nasheeta Peer

**Affiliations:** 1 Non-Communicable Diseases Research Unit, South African Medical Research Council, Cape Town, 7505 and Durban, P.O. Box 19070, Tygerberg, 4091 South Africa; 2 Mental Health, Alcohol, Substances, and Tobacco Research Unit, South African Medical Research Council, Cape Town, 7505 Substances South Africa; 3 Curtin enAble Institute, Faculty of Health Sciences, Curtin University, Perth, WA 6102 Australia; 4 Gender and Health Research Unit, South African Medical Research Council, Cape Town and Pretoria, 7505, 0001 South Africa; 5 School of Public Health and Family Medicine, Faculty of Health Sciences, University of Cape Town, Cape Town, 7925 South Africa; 6 Office of the Executive Scientist, South African Medical Research Council, Cape Town, 7505 South Africa; 7 SAMRC Unit on the Genomics of Brain Disorders, Department of Psychiatry, Faculty of Medicine and Health Sciences, Stellenbosch University, Cape Town, South Africa; 8 Biostatistics Unit, South African Medical Research Council, Cape Town, 7505 South Africa; 9 UNDP-UNFPA-UNICEF-WHO-World Bank Special Programme of Research, Development and Research Training (HRP), Department of Sexual and Reproductive Health and Research, World Health Organization (WHO), Geneva, Switzerland; 10 School of Public Health, University of Witwatersrand, Johannesburg, 2193 South Africa; 11 Department of Medicine, Faculty of Health Sciences, University of Cape Town, Cape Town, 7925 South Africa

**Keywords:** Gender-based violence, Childhood maltreatment, Intimate partner violence, Rape, Mediation analysis, AUDIT-C, Alcohol misuse

## Abstract

**Background:**

The association of traumatic experiences with problematic alcohol use has been described, but data on possible mediation effects of mental distress are sparse. We examined whether mental ill-health mediated the association between trauma exposure across the lifespan and alcohol use.

**Method:**

We analysed cross-sectional data from a sample of rape-exposed and non-rape-exposed women, living in KwaZulu-Natal, with self-reported data on alcohol misuse (AUDIT-C cut-off ≥ 3) and exposure to childhood maltreatment (CM), intimate partner violence (IPV), non-partner sexual violence (NPSV), other traumatic events, and mental ill-health. Logistic regression and multiple mediation models were used to test the mediation effects of symptoms of depression and PTSS on the association between abuse/trauma and alcohol misuse.

**Results:**

Of 1615 women, 31% (n = 498) reported alcohol misuse. Exposure to any CM (adjusted odds ratio (aOR): 1.59, 95% confidence interval (CI): 1.27–1.99), as well as to sexual, physical and emotional CM, were independently associated with alcohol misuse. Lifetime exposure to any IPV (aOR:2.01, 95%CI:1.59–2.54), as well as to physical, emotional and economic IPV, NPSV (aOR: 1.75, 95%CI: 1.32–2.33), and other trauma (aOR:2.08, 95%CI:1.62–2.66), was associated with alcohol misuse. Exposure to an increasing number of abuse types, and other traumatic events, was independently associated with alcohol misuse. PTSS partially mediated the associations of CM, IPV, NPSV and other trauma exposures with alcohol misuse (ps ≤ 0.04 for indirect effects), but depression symptoms did not.

**Conclusions:**

These findings highlight the need for trauma-informed interventions to address alcohol misuse that are tailored to the needs of women who have experienced violence.

**Supplementary Information:**

The online version contains supplementary material available at 10.1186/s13011-023-00549-8.

## Background

In South Africa, recent research suggests that a quarter of women aged 15 years and older consume alcohol and one-third of these binge drink (that is consume ≥ 6 drinks in one sitting) [1]. The social and health burdens associated with alcohol misuse are high in South Africa, with alcohol misuse a major contributor to the burden of infectious disease, non-communicable diseases and injury [2, 3]. Several studies have examined the correlates of alcohol misuse among South African women, noting the importance of contextual drivers of drinking (such as poverty, unemployment, and alcohol availability) [4], as well as psychosocial factors that increase women’s risk of alcohol misuse [5–7]. Notable among these psychosocial factors is exposure to gender-based violence (GBV), either in childhood or adulthood [6, 7]. GBV is highly prevalent among South African women, with a study of 511 women in Gauteng reporting that 50% of women experienced lifetime and 18% past year intimate partner violence (IPV) [8], and 49% with mental health or alcohol-related problems as a result of this violence [8]. South African women urgently need access to interventions that reduce the impact of GBV on their mental health and alcohol use [6, 9]. However, gaps in our understanding of the relationship between GBV, mental health, and alcohol misuse have hampered the design of these interventions.

International studies have shown a link between abuse exposures and alcohol misuse [10–13]. Women exposed to childhood abuse, physical IPV or those who were raped were found to drink more alcohol and were at greater risk for alcohol use disorders compared to women not exposed to this abuse [10, 11]. The relationship between GBV and alcohol misuse may be partially accounted for by post-traumatic stress symptoms (PTSS) and mental health problems that develop as a consequence of this violence exposure [11–13]. Some studies showed that women exposed to GBV such as childhood abuse, rape or physical abuse were more likely to develop post-traumatic stress disorder (PTSD) compared to women not exposed to GBV [14]. PTSS and depression are known to increase risk of alcohol misuse [8, 11, 15–17).

Women report using alcohol to cope with these negative thoughts and emotions [11, 13], with evidence suggesting that drinking to cope mediates the relationship between alcohol misuse and PTSS among survivors of GBV in the USA [11, 18]. While coping motives as a potential mediator of the relationship between PTSS and alcohol use has been extensively explored [19], the extent to which symptoms of post-traumatic stress and depression mediate associations between GBV and alcohol misuse remains under-researched [19, 20]. A better understanding of the pathways through which GBV increases women’s risk of alcohol misuse is needed to identify targets for interventions aimed at reducing alcohol misuse among these vulnerable women.

In addition, associations between GBV and alcohol misuse are likely to vary by type and severity of GBV and type of perpetrator (intimate partner versus non-partner) [21–25], but this has rarely been examined in the literature. Previous studies have shown that symptoms of traumatic stress mediate the relationship between various types of child maltreatment (CM) [26], or cumulative trauma exposure irrespective of type [25], and alcohol use, but these studies have not explored whether the mediating effects of traumatic stress are consistent across CM, IPV and non-partner sexual violence (NPSV) exposures. Understanding how various experiences of GBV affect alcohol misuse may help identify survivors of GBV who are at greater risk for alcohol problems and require prioritisation for these interventions.

This paper responds to these gaps. More specifically, the paper aims to describe (i) the associations between various forms of GBV (CM, IPV, NPSV) and other traumatic experiences (i.e., witnessing a murder, contact crime) and alcohol misuse, and (ii) the extent to which these associations are mediated by symptoms of depression and PTSS in a sample of women aged 18 to 40 years.

## Methods

### Participants and setting

This study analysed baseline data of the Rape Impact Cohort Evaluation (RICE) study which recruited participants in the greater Durban area, KwaZulu-Natal Province, South Africa. The study protocol, published previously, describes the methods in detail [27, 28]. The RICE study recruited two cohorts of women. The rape-exposed cohort comprised women who experienced a rape and presented to the sexual assault service centres within 20 days of the rape incident, regardless of prior exposure to GBV. This time window was chosen as it allowed us to document HIV infection at baseline. This was important as a main aim of the RICE study was to examine HIV acquisition after a rape. Second, we wished to document acute stress reactions to the rape. In the control group, women who have never experienced a rape (non-rape exposed) were recruited from family planning and well-baby clinic services in the same locality as the rape services. Participants were recruited if they were between 18 and 40 years of age. Women who were older than 40 years of age were excluded as we were interested in assessing HIV acquisition and HIV incidence is much lower in this age group relative to younger women [28]. Girls under 16 years were excluded because they constitute a vulnerable group that requires additional ethical safeguards. The women in the control group were screened for exposure to rape or forced sex during adulthood or childhood. Participants were included irrespective of their HIV status. The present study pooled data from both the rape-exposed and non-exposed women for analyses.

### Data collection

Trained research assistants worked with service providers at the recruitment sites to identify eligible participants. A brief introduction of study was done by the service providers and interested women were asked to provide their contact details [28]. The interested women were invited to the RICE study clinic where trained female staff provided more study information and completed consent procedures. Baseline data were collected between August 2014 and March 2019 by trained female staff who administered interview-based questionnaires in the participant’s preferred language (isiZulu, English) using an electronic data capturing and management tool (Bryant System). The interview-administered questionnaires were translated into isiZulu using standard forward and backward translation by research staff and post graduate students respectively.

### Measures

The Alcohol Use Disorders Identification Test-Consumption (AUDIT-C) was used to screen for the presence of alcohol misuse. The AUDIT-C is a short-form of the 10-item AUDIT and has been validated for use in South Africa [29]. The AUDIT-C comprises three items that examine frequency of alcohol consumption, the number of drinks per drinking day and the frequency of binge drinking (defined as consuming ≥ 6 drinks on one occasion) in the 12 months preceding the assessment. Responses to each question are summed to yield total scores ranging from 0 to 12. Higher total scores reflect greater levels of alcohol consumption with cut-offs of 3 or more indicative of alcohol misuse among women [30]. We used this standard cut-off to categorize participants into those with alcohol misuse (scores ≥ 3) and those without (scores < 3). The latter category included participants who reported no or low-risk use.

### Mental health outcomes

***Depressive symptoms***: The 20-item Center for Epidemiologic Studies Depression (CES-D) scale assessed depressive symptoms in the 7 days preceding the interview [31]. Participants responded to these items using a 4-point scale. Responses to items were summed to yield a total score that ranged 0–60. The CES-D has been validated for use in South Africa [32] and extensively used to assess frequency of depressive symptoms in this setting e.g. [33, 34]. In this study we demonstrated high internal reliability of the measure (Cronbach α = 0.92). Higher scores indicate more symptoms of depression. A standard score cut-off of ≥ 16 was used to classify participants into presence of clinically significant symptoms of depression vs. absence of clinically significant symptoms [31].

***Post-traumatic stress symptoms (PTSS)***: The validated Davidson Trauma Scale (self-rated) was used to assess PTSS over the previous week [35, 36]. Responses were evaluated on a five-point scale ranging from ‘not at all’ to ‘all the time’ and summed for an overall PTSS score (Cronbach α = 0.96). A score ≥ 20 defined the presence of PTSS. Since the definition of PTSD includes the presence of traumatic stress responses for longer than four weeks, stress reactions measured in the previous week do not necessarily meet the duration criterion for PTSD.

### Gender-based violence (GBV) exposures

***Childhood maltreatment (CM)***: History of exposure to sexual, physical and emotional abuse/maltreatment, as well as parental neglect before 18 years of age was assessed using the Childhood Trauma Questionnaire Short Form (CTQ-SF) scale [37, 38]. The presence or absence of each CM type was assessed. Overall CM was defined as exposure to any of the four types of CM and categorised as “yes, present” or “no, absent”. Exposure to multiple CM types was classified from zero (no exposure) to four (exposure to all four types of CM). The frequency of exposure to each CM type was categorised as never (1), sometimes (2), often (3), or very often (4). Scores for all four CM types were summed to yield overall scores ranging from 13 to 52. Higher scores indicate greater severity of exposure to CM. In this study, internal reliability for the scale was good (Cronbach α = 0.80).

***Lifetime intimate partner violence (IPV)***: IPV type: The WHO multi-country study questionnaire [39], previously validated in South Africa [40] assessed the frequency of sexual abuse (four items), physical abuse (five items), emotional abuse (seven items) and economic abuse (seven items) by a current or previous intimate partner. In this study, the scale had very high internal consistency (Cronbach α = 0.96). For each IPV type, item responses were summed and then recoded as “yes, present” or “no, not present”. Any IPV was defined as exposure to any of the four types of IPV and categorised as “yes, present,” or “no, not present”. The frequency of exposure to each type of IPV was categorised as never (1), once (2), few (3), many (4). Severity of IPV exposure: The sum of the frequency of exposure for all four IPV types were summed to yield overall scores ranging from 20 to 80, with higher scores indicating greater severity of exposure to IPV. Multiple exposures: Exposure to multiple IPV types was classified from zero (no exposure) to four (exposure to all 4 types of IPV).

***Lifetime non-partner sexual violence (NPSV) exposure***: Four questions explored experiences of sexual violence by non-partners (Cronbach α = 0.70). An affirmative response to any of the four questions was defined as sexual violence by a non-partner. The sum of all frequencies of NPSV exposure determined the overall severity of lifetime NPSV exposure score.

### Lifetime exposure to other traumatic events

The 10-item Life Event Checklist examined exposure to traumatic life events other than childhood maltreatment or gender-based violence in adulthood (Cronbach α = 0.60). Traumatic events that were assessed included imprisonment, witnessing a murder, being robbed at gun or knife point [41]. Each item had a yes (1) or no (0) response option. Item responses were summed for a composite score, with higher scores indicating greater exposure to traumatic events.

### Statistical analysis

Data were analysed using R statistical software version 3.6.0 (2019-04-26). At baseline, the RICE study recruited 1799 women, of whom 184 (10.2%) had missing data on alcohol use, 231 (12.8%) on IPV exposure and one (0.1%) on CM exposure. Participants who were missing data on alcohol use or exposure to GBV were excluded from the analysis, leaving an analytic sample of N = 1615 women. Since this study aimed to examine associations between GBV and alcohol misuse among both rape-exposed and non- exposed women, and not to determine and/or compare the prevalence of GBV and alcohol misuse between the two groups of women, this analysis pooled the baseline data from both cohorts of women. This was motivated by an absence of a significant interaction effect of GBV on the alcohol misuse outcome. This exploratory analysis was done using logistic regression models where the interaction term (CM/IPV/NPSV*rape-exposure) was included in the model for the effects of GBV on alcohol misuse.

Descriptive statistics compared the demographic, socio-economic, and mental health of women with and without alcohol misuse. Baseline characteristics were summarised as means (standard deviation, SD) or medians (interquartile range) for continuous variables, and as counts (percentages) for categorical variables, for the overall sample and by alcohol misuse status. Differences between women who reported alcohol misuse and those who did not, were evaluated using t-tests, Kruskal-Wallis tests or chi-square tests, where appropriate. We conducted two separate sets of analyses where GBV exposures (predictors) were treated as (1) categorical measures (binary and ordinal variables), and (2) as continuous measures. This approach reflects the two ways in which these measures may be used in clinical practice when screening for GBV.

Logistic regression models assessed the association between each GBV variable and the alcohol misuse outcome. Data are presented as odds ratio (OR) and 95% confidence intervals (CI). Separate logistic regression analyses were conducted for each variable of interest (i.e. each abuse type, severity score or mental health outcome). All models were adjusted for potential confounders including age, education, employment, residential area and recent rape exposure. The choice of confounders was based on prior knowledge of their association with the exposure, mediators, and outcome of interest.

We used multiple mediation analyses to explore whether depression and PTSS scores independently mediated associations between CM, IPV, lifetime NPSV, other traumatic exposure variables and the alcohol misuse outcome while adjusting for the potential confounding effects of age, education, employment, residential areas and recent rape exposure. The mediation analyses were conducted using package *Laavan (*http://CRAN.R-project.org/package=lavaan*accessed on 17-September-2021)*. The indirect, direct and total effects (probit regression co-efficients) and the 95% confidence intervals were estimated using the diagonally weighted least squares estimation method. The significance of the mediation effect was tested through bootstrapping 5000 replications with p < 0.05 indicating statistical significance. A complete mediation is present when the total and indirect effects are significant while the direct effect is non-significant. Partial mediation occurs when the total and indirect effects are significant, and the direct effect remains significant. We repeated all analyses in each cohort of women to explore whether the associations were influenced by recent rape exposure.

## Results

Of 1615 women included, 498 (31%) reported alcohol misuse (Table 1). Table 1 presents key participant characteristics by alcohol misuse. There were no significant differences in age, education, employment or income status between the two groups, but participants living in formal urban areas were more likely to meet criteria for alcohol misuse than those in informal areas (77% vs. 69%, p = 0.002). Women meeting criteria for alcohol misuse had higher median depression scores (23 (interquartile range (IQR): 12–37) vs. 18 (IQR: 10–34), p < 0.001] and higher median PTSS scores [21 (IQR: 3–41) vs. 10 (IQR: 0–34), p < 0.001]. They also reported exposure to a higher number of other traumatic events than women who did not report alcohol misuse [2 (IQR: 1–4) vs. 1 (IQR: 0–3), p < 0.001].

**Table 1:** Key participant characteristics by alcohol misuse status

Variables in mean (SD), median (IQR) or n (%)	No-alcohol misuse (n = 1117)	Alcohol misuse (n = 498)	P-value
Age (years)	25.2 (5.6)	25.3 (5.0)	0.668
**Education**, n (%)			0.457
Primary (1–7 years) (n = 651)	443 (39.7)	208 (41.8)	
≥High school (≥ 8 years) (n = 964)	674 (60.3)	290 (58.2)	
**Employment**, n (%)			0.921
No (n = 1285)	890 (79.7)	395 (79.3)	
Yes (n = 330)	227 (20.3)	103 (20.7)	
**Income**, n (%)	**n = 337**	**n = 147**	0.452
<R2000 (n = 339)	233 (69.1)	106 (72.1)	
R2000-R5000 (n = 121)	89 (26.4)	32 (21.8)	
>R5000 (n = 24)	15 (4.5)	9 (6.1)	
**Resident area**, n (%)	**n = 1101**	**n = 493**	0.002
Formal urban	764 (69.4)	378 (76.7)*	
Informal urban	192 (17.4)	77 (15.6)	
Rural and Semi-rural	145 (13.2)	38 (7.7)*	
**Mental health (median, IQR)**			
CES-D depression symptom score (0–60)	18 (10–34)	23 (12–37)*	< 0.001
Post-traumatic stress symptom score (0–68)	10 (0–34)	21 (3–41)*	< 0.001
**Other traumatic exposures score** (0–11)	1 (0–3)	2 (1–4)*	< 0.001

SD, standard deviation; IQR, interquartile range. Alcohol misuse was defined as scored ≥ 3 in the Alcohol Use Disorders Identification Test-Consumption questions (AUDIT-C). No-alcohol misuse as AUDIT-C scored 0–2; Resident areas classification based on understanding where the people live: i.e. formal urban: brick house with available public services such as electricity and tapped water; informal urban: i.e. townships which could be informal housing and where public services are limited; rural and semi-rural: typical farming areas, etc.; CES-D, Center for Epidemiologic Studies Depression: examined experiences of depressive symptoms in the previous week; Post-traumatic stress symptom: measured using Davidson Trauma Scale. Other traumatic exposures: imprisonment, witnessing a murder robbed at gun or knife point, etc. *Indicates a statistical significance

### Associations between GBV exposure with alcohol outcomes

Tables 2 and 3 present bivariate associations between CM, IPV and NPSV and alcohol misuse. A higher proportion of women who met criteria for alcohol misuse reported CM, irrespective of CM type and number of CM types compared to women who did not report alcohol misuse (all ps ≤ 0.05) (Table 2). Women who reported alcohol misuse had higher scores for severity of exposure to all four CM types compared to women who reported no alcohol misuse (all ps ≤ 0.026).

**Table 2:** Childhood maltreatment (CM) exposure by alcohol misuse status

Childhood maltreatment			
CM exposure, n (%)	No alcohol misuse (n = 1116)	Alcohol misuse (n = 498)	P-value
**Any CM**			< 0.001
No (n = 689)	519 (46.5)	170 (34.1)	
Yes (n = 925)	597 (53.5)	328 (65.9)*	
**Sexual abuse**			0.007
No (n = 1413)	994 (89.1)	419 (84.1)	
Yes (n = 201)	122 (10.9)	79 (15.9)*	
**Physical abuse**			0.001
No (n = 949)	686 (61.5)	263 (52.8)	
Yes (665)	430 (38.5)	235 (47.2)*	
**Emotional abuse**			0.008
No (n = 1159)	824 (73.8)	335 (67.3)	
Yes (bn = 455)	292 (26.2)	163 (32.7)*	
**Parental neglect**			0.026
No (n = 1210)	855 (76.6)	355 (71.3)	
Yes (n = 455)	261 (23.4)	143 (28.7)*	
**Exposure to multiple CM types**			< 0.001
0 (n = 689)	519 (46.5)	170 (34.1)*	
1 (n = 413)	265 (23.7)	148 (29.7)*	
2 (n = 281)	189 (16.9)	92 (18.5)	
3 (n = 174)	110 (9.9)	64 (12.8)	
4 (n = 57)	33 (3.0)	24 (4.8)	
**CM severity of exposure score** ^**a**^			
Overall CM, range (13–46)	14 (13–15)	14 (13–16)*	< 0.001
Sexual abuse, mean (SD)	4.2 (0.65)	4.3 (0.78)*	0.026
Physical abuse, range (3–12)	3 (3–12)	3 (3–12)*	< 0.001
Emotional abuse, mean (SD)	3.5 (1.0)	3.7 (1.3)*	0.003
Parental neglect, mean (SD)	3.3 (0.7)	3.5 (1.5)*	0.003

Alcohol misuse was defined as scored ≥ 3 in the Alcohol Use Disorders Identification Test-Consumption questions (AUDIT-C). No-alcohol misuse as AUDIT-C scored 0–2; Any childhood maltreatment (CM): i.e. exposures to sexual, physical or emotional abuse, or parental neglect before 18 years of age was assessed using the Childhood Trauma Questionnaire Short Form (CTQ-SF) scale. Exposure to multiple CM types: any combination of the 4 CM types. CM severity score: frequency of any CM items was added for a total score. ^a^Data are in mean (SD) or median (IQR). *Indicates a statistical significance

**Table 3:** Intimate partner violence and non-partner sexual violence exposure by alcohol misuse status

IPV and NPSV exposure, n (%)	No alcohol misuse (n = 1075)	Alcohol misuse (n = 491)	P-value
**Any IPV**			< 0.001
No (n = 609)	473 (44)	136 (27.7)	
Yes (n = 957)	602 (56)	355 (72.3)*	
**Sexual IPV**			0.088
No (n = 1317)	916 (85.2)	401 (81.7)	
Yes (n = 249)	159 (14.8)	90 (18.3)	
**Physical IPV**			< 0.001
No (n = 805)	617 (57.4)	188 (38.3)	
Yes (n = 761)	458 (42.6)	303 (61.7)*	
**Emotional IPV**			< 0.001
No (n = 854)	627 (58.3)	227 (46.2)	
Yes (n = 712)	448 (41.7)	264 (53.8)*	
**Economic IPV**			0.020
No (n = 1282)	897 (83.4)	385 (78.4)	
Yes (n = 284)	178 (16.6)	106 (21.6)	
**Multiple IPV types**			0.020
0 (n = 609)	371 (60.9)	238 (39.1)*	
1 (n = 325)	124 (38.2)	201 (61.8)*	
2 (n = 321)	135 (42)	186 (58)*	
3 (n = 205)	63 (30.7)	142 (69.3)*	
4 (n = 106)	42 (39.6)	64 (60.4)*	
**IPV severity of exposure score** ^**a**^			
Overall IPV, range (20–80)	21 (20–27)	24 (20–32)*	< 0.001
Sexual IPV, range (4–16)	4 (4–16)	5 (4–16)	0.095
Physical IPV, range (5–20)	5 (5–7)	6 (5–10)*	< 0.001
Emotional IPV, range (7–28)	7 (7–10)	8 (7–13)*	< 0.001
Economic IPV, range (4–16)	4 (4–4)	4 (4–4)*	0.021
**Recent rape**	**n = 1117**	**n = 498**	0.002
No (n = 887)	643 (57.6)	244 (49.0)	
Yes (n = 728)	474 (42.4)	254 (51.0)*	
**Lifetime NPSV**			
Any NPSV	**n = 1117**	**n = 498**	< 0.001
No (n = 1330)	955 (85.5)	375 (75.3)	
Yes (n = 285)	162 (14.5)	123 (24.7)*	
Overall lifetime NPSV severity score, range (4–12)	4.2 (0.7)	4.4 (0.9)*	< 0.001

Alcohol misuse was defined as scored ≥ 3 in the Alcohol Use Disorders Identification Test-Consumption questions (AUDIT-C). No-alcohol misuse as AUDIT-C scored 0–2; Any IPV: Exposure to sexual, physical, emotional or economic IPV. Exposure to multiple IPV types: any combination of the 4 types of IPV. IPV severity score: frequency of any IPV items was added for a total score. Lifetime sexual violence: ever exposed to sexual violence by non-partners since the age of 18 years, excluding the recent rape exposure; Overall lifetime sexual violence severity score: sum of frequency of sexual violence exposure. ^a^Data are in mean (SD) or median (IQR). *Indicates a statistical significance

Similarly, except for sexual IPV, a greater proportion of women reporting alcohol misuse were exposed to all types of IPV (all ps ≤ 0.020), compared to women who reported no alcohol misuse (Table 3). Women who reported alcohol misuse also had greater severity of exposure to overall IPV as well as physical and emotional IPV (all ps < 0.001). Further, women who reported alcohol misuse were more likely to have had a recent rape event (p = 0.002) and to report at least one episode of NPSV (p < 0.001). Similarly, NPSV exposure severity was higher among women who reported alcohol misuse compared to women who reported no alcohol misuse (p < 0.001).

Table 4 presents logistic regression models of GBV exposures on alcohol misuse. Any CM (OR: 1.59; 95%CI: 1.27–1.99), as well as exposure to sexual (OR: 1.42; 95%CI: 1.04–1.94), physical (OR: 1.39; 95%CI: 1.12–1.72) and emotional CM (OR: 1.31; 95%CI: 1.04–1.66), and number of CM types (OR: 1.17; 95%CI: 1.07–1.29) were independently associated with alcohol misuse. Any IPV (OR: 2.01; 95%CI: 1.59–2.54), physical (OR: 2.15; 95%CI: 1.72–2.69), emotional (OR: 1.61; 95%CI: 1.30–2.01) and economic IPV (OR: 1.35; 95%CI: 1.03–1.78), and number of IPV types (OR: 1.26; 95%CI: 1.16–1.37) were independently associated with alcohol misuse after adjusting for potential confounders. When examining associations between severity of exposure to various forms of GBV and alcohol misuse, greater severity of exposure to CM (overall, physical abuse, emotional abuse and parental neglect), IPV (overall, physical and emotional IPV) and NPSV were independently associated with greater odds of alcohol misuse.

**Table 4:** Logistic regression models for the associations of mental health, abuse and other traumatic exposures with alcohol misuse

Alcohol misuse (No alcohol misuse as reference category)				
*Categorical variables*	P-interaction (CM/IPV/NPSV)*RE	AOR	95%CI	P-value
**Mental health**				
Depressive symptoms	0.997	1.31	0.99–1.73	0.060
Post-traumatic stress symptoms	0.347	1.49*	1.10–2.03	0.010
**GBV exposure**				
Any CM	0.046	1.59*	1.27–1.99	< 0.001
Sexual CM	0.068	1.42*	1.04–1.94	0.029
Physical CM	0.306	1.39*	1.12–1.72	0.002
Emotional CM	0.326	1.31*	1.04–1.66	0.022
Parental neglect	0.066	1.27	0.99–1.62	0.057
Multiple CM types (0–4 types)	0.049	1.17*	1.07–1.29	< 0.001
Any IPV	0.424	2.01*	1.59–2.54	< 0.001
Sexual IPV	0.680	1.26	0.94–1.68	0.126
Physical IPV	0.190	2.15*	1.72–2.69	< 0.001
Emotional IPV	0.510	1.61*	1.30–2.01	< 0.001
Economical IPV	0.731	1.35*	1.03–1.78	0.031
Multiple IPV types (0–4 types)	0.215	1.26*	1.16–1.37	< 0.001
Any lifetime NPSV	0.049	1.75*	1.32–2.33	< 0.001
Any other traumatic exposure	0.580	2.08*	1.62–2.66	< 0.001
***Continuous variables***				
**Mental health score**				
CES-D (0–60)	0.943	1.02*	1.01–1.03	0.033
PTSS (0–68)	0.394	1.02*	1.01–1.03	< 0.001
**GBV severity of exposure score**				
Overall CM (13–46)	0.447	1.07*	1.03–1.10	< 0.001
Sexual CM (4–13)	0.477	1.14	0.98–1.32	0.080
Physical CM (3–12)	0.634	1.14*	1.05–1.24	0.001
Emotional CM (3–13)	0.453	1.13*	1.03–1.23	0.009
Parental neglect (3–12)	0.099	1.19*	1.05–1.39	0.005
Overall IPV (20–80)	0.051	1.02*	1.01–1.03	< 0.001
Sexual IPV (4–16)	0.463	1.00	0.94–1.07	0.975
Physical IPV (5–20)	0.003	1.08*	1.05–1.11	< 0.001
Emotional IPV (7–28)	0.415	1.05*	1.02–1.07	< 0.001
Economic IPV (4–16)	0.667	1.03	0.96–1.09	0.444
Lifetime NPSV (4–12)	0.049	1.33*	1.15–1.54	< 0.001
Other traumatic exposures (0–11)	0.035	1.23*	1.15–1.31	< 0.001

Separate logistic regression analyses were conducted for each abuse variable adjusting for age, education, employment, residence and recent rape exposure in all models. Alcohol misuse was defined as scored ≥ 3 in the Alcohol Use Disorders Identification Test-Consumption questions (AUDIT-C). No-alcohol misuse as AUDIT-C scored 0–2. Depressive symptoms: total scores ≥ 16 for CES-D score (Centre for Epidemiologic Studies Depression Scale); total scores ≥ 20 for post-traumatic stress symptoms (PTSS) using Davidson Trauma Scale; Any childhood maltreatment (CM): i.e. exposures to sexual, physical or emotional childhood abuse (CA), or parental neglect before 18 years of age; Any intimate partner violence (IPV): i.e. exposures to sexual, physical, emotional or economic IPV; Any lifetime non-partner sexual violence (NPSV): ever exposed to sexual violence by non-partners since age 18, excluding the recent rape exposure; Any lifetime sexual harassment: experienced unwelcome, inappropriate sexual advances and propositions or threatened or coerced to have sex by a non-partner since age 18; Any other traumatic exposure: exposed to any of imprisonment, witnessing a murder robbed at gun or knife point, etc. CM severity score: frequency of any CM items was added for a total score; multiple CM types: sum of all 4 CM types; IPV severity score: frequency of any IPV items was added for a total score; multiple IPV types: sum of all 4 IPV types; overall lifetime NPSV severity score: sum of frequency of sexual violence exposure; other traumatic exposures: sum of any experiencing imprisonment, witnessing a murder robbed at gun or knife point, etc. *Indicates a statistical significance

Table 5 depicts standardised regression coefficients for the mediating effects of depression and PTSS on associations between exposure to CM, lifetime IPV, lifetime NPSV and other traumatic events and alcohol misuse. The direct effects of any CM, lifetime IPV, lifetime NPSV and other traumatic exposures on alcohol misuse remained significant (all ps ≤ 0.003 for direct effects) and were partially mediated by PTSS scores (all ps ≤ 0.044 for indirect effects). Depression symptom scores did not mediate these associations. In parallel analyses, very similar results were found for the effects of the severity of exposure to CM, IPV and NPSV on alcohol misuse (Table 4). The direct effects of severity of exposure to CM, IPV and NPSV on alcohol misuse were partially mediated by PTSS only (all ps ≤ 0.007 for indirect effects). Neither PTSS nor depression scores (p = 0.294 for indirect effect) mediated the relationship between the number of other traumatic events and alcohol misuse.

**Table 5:** Multiple mediation analyses for the associations of abuse and other traumatic exposures with alcohol misuse (AUDIT-C score > = 3) adjusted for age, level of education, employment, residence and recent rape exposure, in a sample of South African women (N = 1615)

Mediators	Total effect			Direct effect			Indirect effect		
	Co-efficient	95% CI	P-value	Co-efficient	95% CI	P-value	Co-efficient	95% CI	P-value
**Any childhood maltreatment**	0.278*	0.144–0.413	< 0.001	0.225*	0.087–0.364	0.001			
Depression score							0.016	-0.005-0.037	0.133
PTSS score							0.037*	0.010–0.064	0.007
**Any IPV**	0.420*	0.280–0.559	< 0.001	0.377*	0.235–0.520	< 0.001			
Depression score							0.015	-0.007-0.038	0.175
PTSS score							0.027*	0.005–0.048	0.014
**Any lifetime NPSV**	0.346*	0.170–0.523	< 0.001	0.275*	0.093–0.457	0.003			
Depression score							0.021	-0.006-0.047	0.127
PTSS score							0.051*	0.014–0.087	0.007
**Any other traumatic exposure**	0.422*	0.293–0.590	< 0.001	0.383*	0.227–0.593	< 0.001			
Depression score							0.015	-0.010-0.040	0.248
PTSS score							0.044*	0.001–0.087	0.044
**Severity of violence exposure (Continuous measure)**									
Childhood maltreatment score	0.039*	0.017–0.062	0.001	0.029*	0.006–0.053	0.014			
Depression score							0.003	-0.001-0.008	0.139
PTSS score							0.007*	0.002–0.011	0.005
Intimate partner violence (IPV) score	0.014*	0.007–0.020	< 0.001	0.011*	0.004–0.018	0.002			
Depression score							0.001	0.000-0.003	0.155
PTSS score							0.002*	0.001–0.003	0.007
Lifetime NPSV	0.173*	0.095–0.251	< 0.001	0.141*	0.060–0.221	0.001			
Depression score							0.010	-0.002-0.021	0.112
PTSS score							0.023*	0.007–0.039	0.006
Other traumatic event exposures score	0.125*	0.085–0.165	< 0.001	0.109*	0.066–0.152	< 0.001			
Depression score							0.004	-0.004-0.012	0.294
PTSS score							0.012	-0.001-0.026	0.075

Sensitivity analyses demonstrated a similar pattern of findings among women recently exposed to rape (Table S1), although severity of IPV exposure (OR: 1.01; 95%CI: 1.00-1.03; p = 0.057), physical IPV (OR: 1.06; 95%CI: 1.00-1.08; p = 0.054), depression and PTSS scores (both ps ≥ 0.223) were no longer associated with greater odds of alcohol misuse. As depression and PTSS were no longer associated with alcohol misuse, mediation analyses were not conducted in this group. In the non-rape exposed cohort, CM types were not associated with alcohol misuse (all ps ≥ 0.088) (Table S1). Mediation analyses in this cohort revealed that PTSS fully mediated the associations of CM (p = 0.021 for indirect effect; p = 0.384 for direct effect), and partially mediated the association of lifetime NPSV (p = 0.001 for indirect effect; p = 0.009 for direct effect) (Table S2).

## Discussion

This study extends current understandings of the relationship between GBV exposure and alcohol misuse by examining whether symptoms of traumatic stress and depression mediate this relationship, and whether these relationships are consistent for different types of abuse and severity of exposure. Findings suggest (1) a high prevalence of alcohol misuse in this cohort of South African women; (2) positive associations between GBV exposure and alcohol misuse that differ by type, number of types and severity of exposure; and (3) partial mediation by PTSS, but not by depression, of the relationship between GBV exposure and alcohol misuse.

Our findings highlight associations between various forms of GBV in childhood and adulthood, and women’s alcohol misuse. Similar to findings from a national study in the US [42], all types of CM except for parental neglect were associated with greater odds of alcohol misuse. This finding contrasts with earlier South African studies that reported associations between child sexual abuse, but not other types of childhood violence experiences, and problem drinking in rural South African youth [38]; and no association of CM with risk of alcohol use disorder among university students [7]. Some of these differences might be explained by these studies possibly being underpowered to detect associations between alcohol misuse and GBV due to low rates of alcohol misuse in the study samples, smaller sample sizes, and missing data. Our finding of significant associations between exposure to multiple CM types and alcohol misuse is in keeping with those of a systematic review that showed greater odds of problem alcohol use among participants with multiple adverse childhood experiences (≥ 4 types) [43].

This study also found associations of severity of exposure to overall IPV, and physical and emotional IPV with alcohol misuse. This is novel as earlier studies mainly examined physical or sexual IPV, with little focus on emotional or economic IPV [5, 6, 44] The finding that economic IPV is not significantly associated with increased alcohol misuse is not surprising given that economic IPV is likely to limit women’s ability to purchase alcohol. This explanation however warrants further research. In addition, we found significant associations between NPSV and alcohol misuse. The finding that both sexual abuse during childhood and sexual IPV were not associated with alcohol misuse also needs further exploration. Globally this is the least reported form of GBV and this could likely be linked to under reporting [45].

Importantly, our findings suggest that PTSS but not depression partially mediates the relationship between alcohol misuse and exposure to any CM, IPV and NPSV, exposure to other traumatic experiences, as well as severity of these exposures. These findings suggest that symptoms of traumatic stress may partially account for the relationship between traumatic experiences in childhood and adulthood and women’s use of alcohol. While other studies have shown that symptoms of traumatic stress mediate the relationship between various types of CM [26], or cumulative trauma exposure irrespective of type [25], and alcohol use, these studies have not looked at whether the mediating effects of traumatic stress are consistent across all CM, IPV and NPSV exposures. Our study extends these earlier findings to demonstrate that PTSS partially mediates the relationship between GBV exposure and alcohol misuse, irrespective of whether this exposure occurred in childhood or later in life, or whether it was perpetrated by an intimate partner or non-partner. These findings suggest that addressing PTSS symptoms should be an important part of interventions to reduce alcohol misuse among survivors of any form of GBV. Qualitative research with trauma-exposed South African women who use alcohol supports the need for trauma-informed interventions to address alcohol misuse that combine evidence-based psychological interventions for PTSS with cognitive-behavioral strategies to support alcohol reduction [46]. More research is needed to understand why depressive symptoms did not mediate the relationship between GBV and alcohol misuse. It could be that women in the target communities mainly consumed alcohol in social environments outside of their homes, like taverns and shebeens. Women with symptoms of depression may be less likely to frequent these venues to consume alcohol.

Several limitations and sources of bias should be considered when interpreting these findings. First, the data are cross-sectional, and we do not have information about the timing of these GBV exposures. As such, we cannot make claims about the temporality of these relationships. Alcohol misuse among women is stigmatized in South Africa [47] and this may have led to under-reporting of consumption and the subsequent misclassification of women into alcohol misuse or non-misuse categories. Another limitation is that the sample included two groups of women who were recruited voluntarily at post-rape service centres (rape exposed group) and primary health care facilities (control group), and thus do not represent all women who were raped or those who seek care post rape or the general population of women. Apart from this recent rape exposure, we assumed the two groups to be similar in that they were both active health service users. We also controlled for the experience of rape in all our analyses. We also acknowledge the potential for Type 1 error in our analyses given the many comparisons, highlighting the importance of replicating these findings.

## Conclusions

This cross-sectional study found that CM, IPV, NPSV and other traumatic exposures were positively associated with alcohol misuse among South African women. The associations were partially mediated by symptoms of PTSS, but not by symptoms of depression. These findings highlight the need for trauma-informed interventions to address alcohol misuse that are tailored to the needs of women who have experienced violence.

## Electronic supplementary material

Below is the link to the electronic supplementary material.

Supplementary Material 1

## Data Availability

The datasets used and/or analysed during the current study are available from the corresponding author on reasonable request.
