# Supplementary material for: Symptoms of posttraumatic stress partially mediate the relationship between gender-based violence and alcohol misuse among South African women
Source: Subst Abuse Treat Prev Policy. 2023 Jun 22;18:38. doi: 10.1186/s13011-023-00549-8 (PMC10288665; doi:10.1186/s13011-023-00549-8)
Supplement: Supplementary file 1 — Supplementary Material 1 [file 13011_2023_549_MOESM1_ESM.docx]

Supplementary Table legends

Supplementary Tables

**Table S1: Logistic regression models for the associations of mental health, abuse and other traumatic exposures with alcohol misuse in non-rape exposed women and recent rape exposure using no-alcohol misuse as reference category.**

| **Non-rape exposed group (N=887)** | | | | **Recent rape exposure group (N=728)** | | | |
| --- | --- | --- | --- | --- | --- | --- | --- |
| ***Categorical variables*** | **AOR** | **95%CI** | **P-value** | ***Categorical variables*** | **AOR** | **95%CI** | **P-value** |
| **Mental health** |  |  |  | **Mental health** |  |  |  |
| Depressive symptoms | 1.30 | 0.95-1.79 | 0.102 | Depressive symptoms | 1.35 | 0.77-2.47 | 0.307 |
| PTSS | 1.72* | 1.11-2.64 | 0.013 | PTSS | 1.31 | 0.86-2.00 | 0.223 |
| **Abuse/trauma exposure** |  |  |  | **Abuse/trauma exposure** |  |  |  |
| Any CM | 1.30 | 0.96-1.76 | 0.088 | Any CM | 2.05* | 1.47-2.87 | <0.001 |
| Sexual CM | 0.98 | 0.55-1.67 | 0.943 | Sexual CM | 1.72* | 1.16-2.55 | 0.006 |
| Physical CM | 1.24 | 0.91-1.67 | 0.167 | Physical CM | 1.59* | 1.16-2.18 | 0.003 |
| Emotional CM | 1.20 | 0.86-1.67 | 0.283 | Emotional CM | 1.48* | 1.05-2.06 | 0.022 |
| Parental neglect | 1.00 | 0.70-1.43 | 0.984 | Parental neglect | 1.58* | 1.13-2.23 | 0.008 |
| Multiple CM types (0-4) | 1.07 | 0.94-1.22 | 0.301 | Multiple CM types (0-4) | 1.28* | 1.13-1.45 | <0.001 |
| Any IPV | 2.27* | 1.65-3.61 | <0.001 | Any IPV | 1.77* | 1.26-2.51 | 0.001 |
| Sexual IPV | 1.37 | 0.88-2.11 | 0.158 | Sexual IPV | 1.20 | 0.81-1.78 | 0.359 |
| Physical IPV | 2.59* | 1.90-3.54 | <0.001 | Physical IPV | 1.81* | 1.31-2.50 | <0.001 |
| Emotional IPV | 1.73* | 1.28-2.35 | <0.001 | Emotional IPV | 1.52* | 1.10-2.01 | 0.010 |
| Economical IPV | 1.47 | 0.99-2.16 | 0.051 | Economical IPV | 1.27 | 0.86-1.88 | 0.231 |
| Multiple IPV types (0-4) | 1.35* | 1.20-1.53 | <0.001 | Multiple IPV types (0-4) | 1.20* | 1.06-1.35 | 0.003 |
| Any lifetime NPSV | 2.74* | 1.51-4.98 | 0.001 | Any lifetime NPSV | 1.52* | 1.10-2.11 | 0.012 |
| Any other traumatic exposure | 2.26* | 1.64-3.12 | <0.001 | Any other traumatic exposure | 1.96* | 1.33-2.92 | <0.001 |
| ***Continuous variables*** |  |  |  | ***Continuous variables*** |  |  |  |
| **Mental health score** |  |  |  | **Mental health score** |  |  |  |
| CES-D (0-60) | 1.01 | 1.00-1.03 | 0.173 | CES-D (0-60) | 1.01 | 1.00-1.02 | 0.099 |
| PTSS (0-68) | 1.02* | 1.01-1.03 | 0.005 | PTSS (0-68) | 1.02* | 1.01-1.03 | 0.015 |
| **GBV exposure severity score** |  |  |  | **GBV exposure severity score** |  |  |  |
| Overall CM (13-46) | 1.04 | 0.98-1.11 | 0.209 | Overall CM (13-46) | 1.08* | 1.03-1.13 | 0.001 |
| Sexual CM (4-13) | 1.01 | 0.67-1.45 | 0.972 | Sexual CM (4-13) | 1.16 | 0.99-1.38 | 0.066 |
| Physical CM (3-12) | 1.11 | 0.98-1.25 | 0.107 | Physical CM (3-12) | 1.17* | 1.04-1.31 | 0.007 |
| Emotional CM (3-13) | 1.08 | 0.91-1.27 | 0.373 | Emotional CM (3-13) | 1.16* | 1.04-1.29 | 0.010 |
| Parental neglect (3-12) | 1.03 | 0.83-1.26 | 0.788 | Parental neglect (3-12) | 1.29* | 1.10-1.52 | 0.001 |
| IPV (20-80) | 1.04* | 1.02-1.06 | <0.001 | IPV (20-80) | 1.01 | 1.00-1.03 | 0.057 |
| Sexual IPV (4-16) | 1.05 | 0.93-1.17 | 0.414 | Sexual IPV (4-16) | 0.98 | 0.91-1.06 | 0.660 |
| Physical IPV (5-20) | 1.14* | 1.09-1.19 | <0.001 | Physical IPV (5-20) | 1.04 | 1.00-1.08 | 0.054 |
| Emotional IPV (7-28) | 1.06* | 1.02-1.10 | 0.001 | Emotional IPV (7-28) | 1.04* | 1.01-1.07 | 0.012 |
| Economical IPV (4-16) | 1.02 | 0.90-1.13 | 0.793 | Economical IPV (4-16) | 1.04 | 0.95-1.12 | 0.405 |
| Lifetime NPSV (4-12) | 2.00* | 1.29-3.19 | 0.002 | Lifetime NPSV (4-12) | 1.26* | 1.08-1.47 | 0.003 |
| Other traumatic exposures (0-11) | 1.34* | 1.21-1.49 | <0.001 | Other traumatic exposures (0-11) | 1.15* | 1.05-1.25 | 0.002 |

Separate logistic regression analyses were conducted for each abuse variable adjusting for age, education, employment and residence in all models. Alcohol misuse was defined as scored ≥3 in the Alcohol Use Disorders Identification Test-Consumption questions (AUDIT-C). No-alcohol misuse as AUDIT-C scored 0-2. Depressive symptoms: total scores ≥16 for CES-D score (Centre for Epidemiologic Studies Depression Scale); total scores ≥20 for post-traumatic stress symptoms (PTSS) using Davidson Trauma Scale; Any childhood maltreatment (CM): i.e. exposures to sexual, physical or emotional childhood abuse (CA), or parental neglect before 18 years of age; Any intimate partner violence (IPV): i.e. exposures to sexual, physical, emotional or economic IPV; Any lifetime non-partner sexual violence (NPSV): ever exposed to sexual violence by non-partners since age 18, excluding the recent rape exposure; Any lifetime sexual harassment: experienced unwelcome, inappropriate sexual advances and propositions or threatened or coerced to have sex by a non-partner since age 18; Any other traumatic exposure: exposed to any of imprisonment, witnessing a murder robbed at gun or knife point, etc. CM severity score: frequency of any CM items was added for a total score*;* multiple CM types: sum of all 4 CM types; IPV severity score: frequency of any IPV items was added for a total score; multiple IPV types: sum of all 4 IPV types; overall lifetime NPSV severity score: sum of frequency of sexual violence exposure; other traumatic exposures: sum of any experiencing imprisonment, witnessing a murder robbed at gun or knife point, etc. *Indicates a statistical significance.

**Table S2: Multiple mediation analyses for the associations of abuse and other traumatic exposures with alcohol misuse (AUDIT-C score>=3) adjusted for age, level of education, employment and residence, in non-rape exposed women (N=887)**

| **Mediators** | **Total effect** | | | | **Direct effect** | | | **Indirect effect** | | | |
| --- | --- | --- | --- | --- | --- | --- | --- | --- | --- | --- | --- |
|  | Co-efficient | 95% CI | P-value | Co-efficient | | 95% CI | P-value | | Co-efficient | 95% CI | P-value |
| **Any childhood maltreatment** | 0.156 | -0.025-0.337 | 0.091 | 0.084 | | -0.105- 0.274 | 0.384 | |  |  |  |
| Depression score |  |  |  |  | |  |  | | 0.019 | -0.016-0.054 | 0.286 |
| PTSS score |  |  |  |  | |  |  | | 0.053* | 0.008-0.098 | 0.021 |
| **Any IPV** | 0.488* | 0.297-0.678 | <0.001 | 0.448* | | 0.250-0.647 | <0.001 | |  |  |  |
| Depression score |  |  |  |  | |  |  | | 0.055 | -0.039-0.048 | 0.823 |
| PTSS score |  |  |  |  | |  |  | | 0.034 | -0.005-0.074 | 0.090 |
| **Any lifetime NPSV** | 0.625* | 0.256-0.993 | 0.001 | 0.512* | | 0.130-0.895 | 0.009 | |  |  |  |
| Depression score |  |  |  |  | |  |  | | 0.028 | -0.044-0.100 | 0.443 |
| PTSS score |  |  |  |  | |  |  | | 0.084* | 0.004-0.165 | 0.001 |
| **Any other traumatic exposure** | 0.494* | 0.299-0.689 | <0.001 | 0.456* | | 0.234-0.678 | <0.001 | |  |  |  |
| Depression score |  |  |  |  | |  |  | | 0.007 | -0.036-0.051 | 0.737 |
| PTSS score |  |  |  |  | |  |  | | 0.031 | -0.067-0.129 | 0.540 |
| **Severity of violence exposure (Continuous measure)** |  |  |  |  | |  |  | |  |  |  |
| Childhood maltreatment score | 0.025 | -0.016-0.065 | 0.231 | 0.007 | | -0.036-0.050 | 0.749 | |  |  |  |
| Depression score |  |  |  |  | |  |  | | 0.005 | -0.004-0.015 | 0.274 |
| PTSS score |  |  |  |  | |  |  | | 0.012* | 0.002-0.022 | 0.015 |
| Intimate partner violence (IPV) score | 0.023* | 0.013-0.034 | <0.001 | 0.020* | | 0.009-0.032 | <0.001 | |  |  |  |
| Depression score |  |  |  |  | |  |  | | 0.001 | -0.003-0.004 | 0.861 |
| PTSS score |  |  |  |  | |  |  | | 0.003 | 0.000-0.005 | 0.091 |
| Lifetime NPSV score | 0.414* | 0.196-0.632 | <0.001 | 0.335* | | 0.105-0.564 | 0.004 | |  |  |  |
| Depression score |  |  |  |  | |  |  | | 0.021 | -0.030-0.071 | 0.420 |
| PTSS score |  |  |  |  | |  |  | | 0.059* | 0.005-0.113 | 0.033 |
| Other traumatic event exposures score | 0.180* | 0.119-0.241 | <0.001 | 0.178* | | 0.107-0.249 | <0.001 | |  |  |  |
| Depression score |  |  |  |  | |  |  | | 0.001 | -0.014-0.015 | 0.910 |
| PTSS score |  |  |  |  | |  |  | | 0.001 | -0.032-0.034 | 0.956 |

*Indicates a statistical significance.
